# The potential oncogenic effect of tissue-specific expression of JC polyoma T antigen in digestive epithelial cells

**DOI:** 10.1007/s11248-023-00352-y

**Published:** 2023-05-29

**Authors:** Hua-chuan Zheng, Hang Xue, Hong-zhi Sun, Wen-jing Yun, Zheng-guo Cui

**Affiliations:** 1 Department of Oncology and Central Laboratory, The Affiliated Hospital of Chengde Medical University, Chengde, 067000 China; 2 Cancer Center, The First Affiliated Hospital of Jinzhou Medical University, Jinzhou, 121001 China; 3 Department of Environmental Health, University of Fukui School of Medical Sciences, Fukui, 910-1193 Japan

**Keywords:** Gastroenterological cancers, John Cunningham virus T antigen, Oncogenesis, Transgenic mouse

## Abstract

JC polyoma virus (JCPyV), a ubiquitous polyoma virus that commonly infects people, is identified as the etiologic factor for progressive multifocal leukoencephalopathy and has been closely linked to various human cancers. Transgenic mice of CAG-loxp-Laz-loxp T antigen were established. T-antigen expression was specifically activated in gastroenterological target cells with a LacZ deletion using a cre-loxp system. Gastric poorly-differentiated carcinoma was observed in T antigen-activated mice using K19-cre (stem-like cells) and PGC-cre (chief cells), but not Atp4b-cre (parietal cells) or Capn8-cre (pit cells) mice. Spontaneous hepatocellular and colorectal cancers developed in Alb-cre (hepatocytes)/T antigen and villin-cre (intestinal cells)/T antigen transgenic mice respectively. Gastric, colorectal, and breast cancers were observed in PGC-cre/T antigen mice. Pancreatic insulinoma and ductal adenocarcinoma, gastric adenoma, and duodenal cancer were detected in Pdx1-cre/T antigen mice. Alternative splicing of T antigen mRNA occurred in all target organs of these transgenic mice. Our findings suggest that JCPyV T antigen might contribute to gastroenterological carcinogenesis with respect to cell specificity. Such spontaneous tumor models provide good tools for investigating the oncogenic roles of T antigen in cancers of the digestive system.

## Introduction

The JC polyoma virus (JCPyV), together with simian 40 (SV40) and BK viruses, belongs to the human non-enveloped polyomavirus family and is considered an established etiologic factor of progressive multifocal leukoencephalopathy (PML) (Imperiale 2000; Reiss and Khalili 2003). Of JCPyV genes, an early gene encodes T antigen. As an important oncogenic and multifunctional phosphoprotein, T antigen is essential for viral DNA replication because it binds to and unwinds the double helix of DNA and recruits ATPase, helicase, and polymerase (Khalili et al. 2006; Zheng et al. 2022). In the nucleus, T antigen can inactivate p53 and pRb proteins to speed up the cell cycle and subsequently proliferation. In the cytoplasm, T antigen suppresses the Wnt pathway by targeting βTrCP1/2 leading to abnormal β-catenin degradation, and disrupts the insulin growth factor-1 receptor signaling pathway (Zheng et al. 2009; Prado et al. 2018). Additionally, T antigen down-regulates Bag-3 expression in the suppression of apoptosis by blocking AP2 binding to the Bag3 promoter (Sariyer et al. 2012).

JCPyV is a neurotropic virus in the absence of an appropriate immune response, such as with the use of immunosuppressive drugs or AIDS, because its replication is dependent on transcriptional factors in glial cells and neurons, including Jun, NF-1, GF-1, Sp1, Sμbp-2, Purα, and YB-1. However, the virus enters tonsillar tissue from the inhalation of air droplets, and persists quiescent in lymphoid and renal tissues during latency (Wang et al. 2012). Hence, higher copies and positive rate of T antigen DNA were detected in oral, esophageal, gastric, colorectal, anal, head neck squamous carcinoma, lung cancer, prostatic cancer, cervical cancer, and urothelial carcinoma (Khalili et al. 2006; Murai et al. 2007; Zheng et al. 2007a, b, 2009; Kutsuna et al. 2008; Gjoerup and Chang 2010; Wang et al. 2012; Anzivino et al. 2015; Shavaleh et al. 2020).

Intravenous or intracranial inoculation of JCPyV into mice can result in the development of glioblastomas, astrocytomas, medulloblastomas, and neuroblastomas. The transgenic mouse with an early encoding region of the archetype strain of JCPyV was generated using its own promoter. This mouse developed neural crest tumors, such as primitive neuroectodermal tumors, adrenal neuroblastomas, medulloblastomas, pituitary tumors, glioblastomas, malignant peripheral nerve sheath tumors, primitive neuroectodermal tumors, and pituitary tumors (Khalili et al. 2006; Zheng et al. 2009, 2022; Dalianis and Hirsch 2013; Prado et al. 2018). Recently, we established transgenic mice and found that T antigen induced lens tumors and lung cancer, which provided another direct evidence for the potential oncogenic role of JCPyV (Noguchi et al. 2013; Gou et al. 2015). Subsequently, we for the first time found that the insertion of T antigen into the genome could cause lung cancers and lens tumors, but alternative splicing of its intron did not show cell specificity (Gou et al. 2015). To clarify the potential oncogenic roles of JCPyV in cancers of the digestive system, we firstly generated a CAG-loxp-LacZ-loxp-T antigen transgenic mouse. We activated T-antigen expression in gastric, intestinal, pancreatic ductal epithelial, and islet β-cells or hepatocytes using various cre tool mice, and observed spontaneous carcinogenesis with the detection of the alternative splicing of T antigen.

## Materials and methods

### Animal model

pBS-JCPyV Mad1 (kindly provided by Prof. Hirofumi Sawa, Hokkaido University), K19-COX-2 (kindly provided by Prof. Masanobu Oshima, Kanazawa University), and PBS-cre (kindly provided by Prof. Zhi-hong Zheng, China Medical University) were used for CAG-loxp-LacZ T antigen (including intron), K19-cre, and PGC-cre mice at the Shanghai Biomodel Organism Science & Technology Development Co. Ltd. To activate T-antigen expression in gastric, intestinal, pancreatic ductal epithelial, and islet β-cells or hepatocytes, we crossed CAG-loxp-LacZ T antigen mice with Atp4b-cre (gastric parietal cells, kindly provided by Prof. Xiao Yang, Genetic Laboratory of Development and Diseases, Institute of Biotechnology, Beijing, China), Capn8-cre (gastric pit cells, also kindly provided by Prof. Xiao Yang), PGC-cre (gastric chief cells, unpublished), K19-cre (gastric stem-like cells), villin-cre (intestinal epithelium, Jax Lab), Pdx1-cre (pancreas, stomach and duodenum, Jax Lab), and Alb-cre (hepatocytes, Jax Lab) mice. The mice were kept under specific pathogen-free conditions and their characteristics are outlined in Table 1. All procedures and housing were approved by Animal Experiments Committee of The Affiliated Hospital of Chengde Medical University.

**Table 1:** The summary of transgenic mouse strains

Mouse strains	Cells expressing cre	Tumorigenic site
Alb-cre/JCPyV T antigen	Hepatocytes	Liver
Villin-cre/JCPyV T antigen	Intestinal cells	Intestines
PGC-cre/JCPyV T antigen	Chief cells	Stomach; breast
Pdx1-cre/JCPyV T antigen	Pancreas, stomach and duodenum	Pancreas; duodenum

### PCR

We extracted DNA from mouse tails and tissues using a proteinase K/phenol/chloroform method. DNA was amplified by PCR targeting of the T antigen (Forward: 5′-TGGCCTGTAAAGTTCTAGGCA -3′ and Reverse: 5′-GCAGAGTCAAGGGATTTACCTTC-3′), cre (Forward: 5′-GCCTGCATTACCGGTCGATGC-3′ and Reverse: 5′-CAGGGTGTTATAAGCAATCCC-3′), and LaZ (Forward: 5′-AAAGTCGTCCTGAGTTGTTAT -3′ and Reverse: 5′-GCGAAGAGTTTGTCCTCAACC-3′). Total RNA was extracted from the normal tissues of transgenic mice using Trizol reagent (Invitrogen, Waltham, MA, USA; Cat. No. 15596026) and used for cDNA synthesis. To confirm the alternative splicing of T antigen mRNA and the correct splicing of T antigen, we designed primers (Forward: 5′-TCATCATCACTGGCAAAC-3′ and Reverse: 5′-GCAAAGAACTCCACCCT-3′) as previously documented (Gou et al. 2015).

### Western blot

We extracted protein from cell lysates in radio-immunoprecipitation assay lysis buffer, separated proteins in a 10% polyacrylamide gel by SDS polyacrylamide gel electrophoresis, and transferred proteins to a polyvinylidene fluoride membrane (GVS, Bologna, Italy; Cat. No. 1212639). After blocking the membrane in 5% milk (Beyotime Biotechnology, Haimen, China; Cat. No. P0216-300 g) in TBST (Tris buffer solution with tween-20, 20 mmol/L Tris–Cl pH7.5, 500 mmol/L NaCl, 0.05% tween-20), it was incubated with mouse anti-SV40 T antigen (Abcam, Cambridge, UK; Cat. No. ab16879), rabbit anti-PGC (Pepsinogen C, Proteintech, Chicago, IL, USA; Cat. No. 28532-1-AP) or mouse anti-GAPDH antibodies (Proteintech; Cat. No. 60004-1-Ig) in TBST, and then with anti-mouse (Bioss; Cat. No. bs-0296G-biotin) or anti-rabbit IgG conjugated to horse radish peroxidase (HRP; Bioss, Woburn, MA, USA; Cat. No. bs-0295G-Biotin). Bands were visualized with an Azure Biosystem C300 by ECL detection kit (NCM Biotech, Newport, RI, USA; Cat. No. P10300).

### Enzyme-linked immunosorbent assay

An enzyme-linked immunosorbent assay (ELISA) was employed to quantify the serum PGC I level (Abcam; Cat. No. ab275552). Briefly, we incubated either a standard or serum sample in anti-PGC I-antibody-coated polystyrene plates for 1 h. After removing liquid, we added biotin-antibody working solution to each well and incubated the plate for 2 h. After aspiration from each well and three rinses with 350 μL wash buffer, we added 100 μL HRP-avidin working solution to each well and incubated for 1 h. After washing three times, we added 90 μL of TMB (3, 3′, 5, 5′—tetramethylbenzidine) to the wells of the plates, and incubated these for 30 min. Finally, we dispensed stopping solution to each well and measured the absorbance.

### Computed tomography

A Bruker computed tomography (CT) scanner (SkyScan1276) was used to image spontaneous tumors. In brief, mice were put onto the polystyrene bed of a CT scanner. The images were taken with the scan set of the X-ray source: 20.19-μm image pixel size, 388-ms exposure, 0.5-mm filter Al, rotation step (deg) = 0.400, use 360 Rotation, 70 kV tube voltage, 200 μA tube current, and 1344 × 2016 matrix. The NRecon reconstruction conditions of minimum and maximum image conversions for CT scanning = 0.000000 and 0.050000, respectively.

### Patients

Gastric cancer (n = 358) and normal mucosa (n = 130, more than 4 cm from the cancer), and breast cancer (n = 219) and normal tissues (n = 43, more than 4 cm from the cancer) were sampled at The First Affiliated Hospital of Jinzhou Medical University. Tissues were taken from patients who had never received adjuvant treatment, radiotherapy or chemotherapy before their operation. Patients gave written informed consent and the ethics committees of our hospital approved the study.

### Histopathology

We fixed tissues in 4% formaldehyde, embedded these in paraffin and cut them into 4 μm-thick sections. Mouse spontaneous tumors were histologically diagnosed according to hematoxylin- and-eosin (HE) staining in transgenic mice expressing the T antigen of JCPyV. All human tissues were subjected to tissue microarray preparation. Consecutive sections were deparaffinized with xylene, rehydrated with alcohol, and subjected to immunohistochemistry as previously reported (Zhao et al. 2017). Most of the following biomarkers were used to perform histological and molecular diagnoses of spontaneous tumors from transgenic mice. Anti-SV40 T antigen antibody was purchased from Abcam (Cat. No. ab16879), anti-PGC from Proteintech (Cat. No. 28532-1-AP), and anti-pancreas/duodenum homeobox protein 1 (Pdx; Cat. No. ab219207), anti-cytokeratin 19, (CK19; Cat. No. ab52625), anti-ki-67 (Cat. No. ab15580), anti-caudal type homeobox 2 (CDX2; Cat. No. ab76541), anti-villin (Cat. No. ab97512), anti-CEA (Cat. No. ab133633), and anti-mucin 1 (MUC1; Cat. No. ab109185) were from Abcam.

### Bioinformatics analysis

The expression data (RNA-seqV2) of gastric and breast cancers, and their normal tissues were downloaded from The Cancer Genome Atlas (TCGA) database by TCGA-assembler in R software. We integrated the raw data and analyzed CK19 and PGC mRNA expression.

### Statistical analysis

A *Fisher* test was used to compare positive rates of protein expression, and a student *t* test to compare means. We employed SPSS 10.0 software for data analysis. *P* < 0.05 was considered as statistically significant.

## Results

### T antigen was alternatively spliced and overexpressed in transgenic mice

**Fig. 1:**
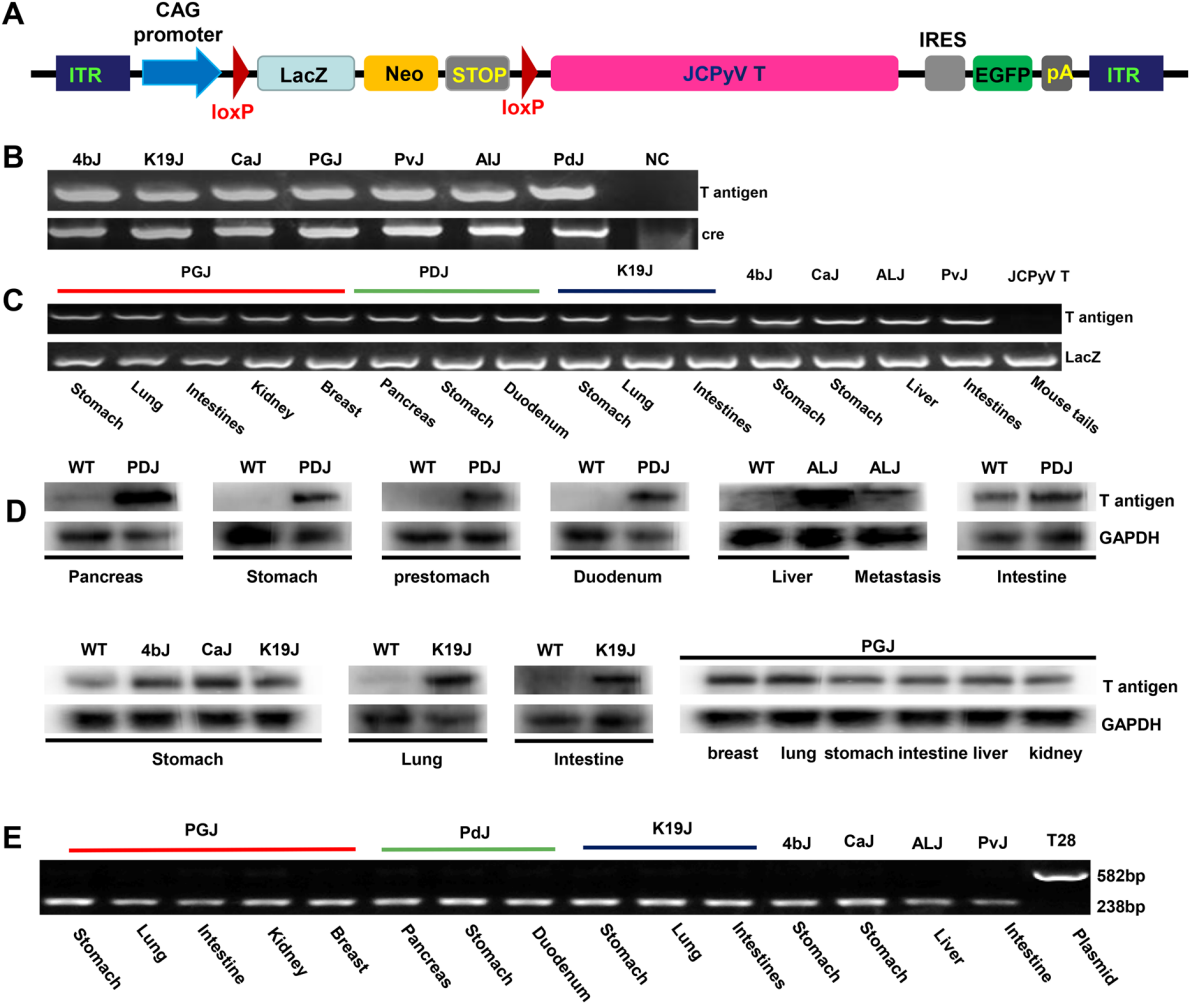
The generation and characterization of JCPyV T antigen transgenic mice. CAG-loxp-Laz-loxp-T antigen transgenic mice were established using a CAG promoter as outlined in the schematic diagram. To activate tissue-specific expression of T antigen, the mice were crossed with Atp4b-cre (gastric parietal cells), PGC-cre (gastric chief cells), Capn8-cre (gastric pit cells), K19-cre (stem-like cells), villin-cre (intestinal epithelial cells), Alb-cre (hepatocytes), and Pdx1-cre (pancreas and gastrointestinal cells) mice (**A**). These positive mice showed cre + /T antigen + after the PCR of tail DNA (**B**). To verify the successful knockout of LaZ, we performed PCR on the DNA of promoter-specific organs targeting the LacZ and CAG/T antigen (**C**). T-antigen expression was examined in target organs of transgenic mice by western blot (**D**). Finally, RT-PCR was employed to detect alternative splicing of the T antigen intron using the mRNA of target organs in transgenic mice (**E**). AlJ, Alb-cre/ JCPyV T antigen; CaJ, Capn8-cre/JCPyV T antigen; IRES, internal ribosome entry site; ITR, inverted terminal repeat; K19J, K19-cre/JCPyV T antigen; NC, negative control; PdJ, Pdx1-cre/JCPyV T antigen; PGJ, PGC-cre/JCPyV T antigen; PvJ, villin-cre/CPyV T antigen; RT-PCR, reverse transcription PCR; T28, PBS-T antigen plasmid as positive control; WT, wild-type; 4bJ, Atp4b-cre/JCPyVT antigen

Transgenic mice with CAG-loxp-LacZ-loxp T antigen were established (Fig. 1A). To activate the expression of T antigen in gastric parietal, chief, stem-like, pancreatic duct, islet β-, and intestinal cells as well as hepatocytes, the mice were crossed with Atp4b-cre, PGC-cre (E et al. 2023), Capn8-cre, K19-cre, Alb-cre, Pdx1-cre, and villin-cre mice, respectively. We employed tail DNA PCR to screen positive mice for the presence of cre and T antigen (Fig. 1B). We used target organ DNA PCR to verify the successful activation of T antigen targeting LacZ (Fig. 1C). Western blots revealed that T antigen was strongly expressed in stomach, pre-stomach, duodenum, intestine, pancreas, liver, breast, lung, kidney, and metastatic cancer tissues of these transgenic mice, especially those expressing T antigen in target organs (Fig. 1D). To analyze the alternative splicing of T antigen and confirm the correct transcription of T antigen, we designed primers targeting the T antigen intron as previously described (Gou et al. 2015). Intron deletion was found in cDNA samples from the stomach, lung, intestine, kidney, breast of the PGC-cre/T antigen mouse; the pancreas, stomach and duodenum of the Pdx1-cre/T antigen mouse; the stomach, lung and intestine of the K19-cre/T antigen mouse; the stomach of Atp4b/T antigen and Capn8-cre/T antigen mice; the liver of the Alb-cre/T antigen mouse; and the intestine of the villin-cre/T antigen mouse (Fig. 1E), suggesting that T antigen showed the correct transcription in these transgenic mice.

### Gastric and colorectal neoplasia was observed in T-antigen transgenic mice

**Fig. 2:**
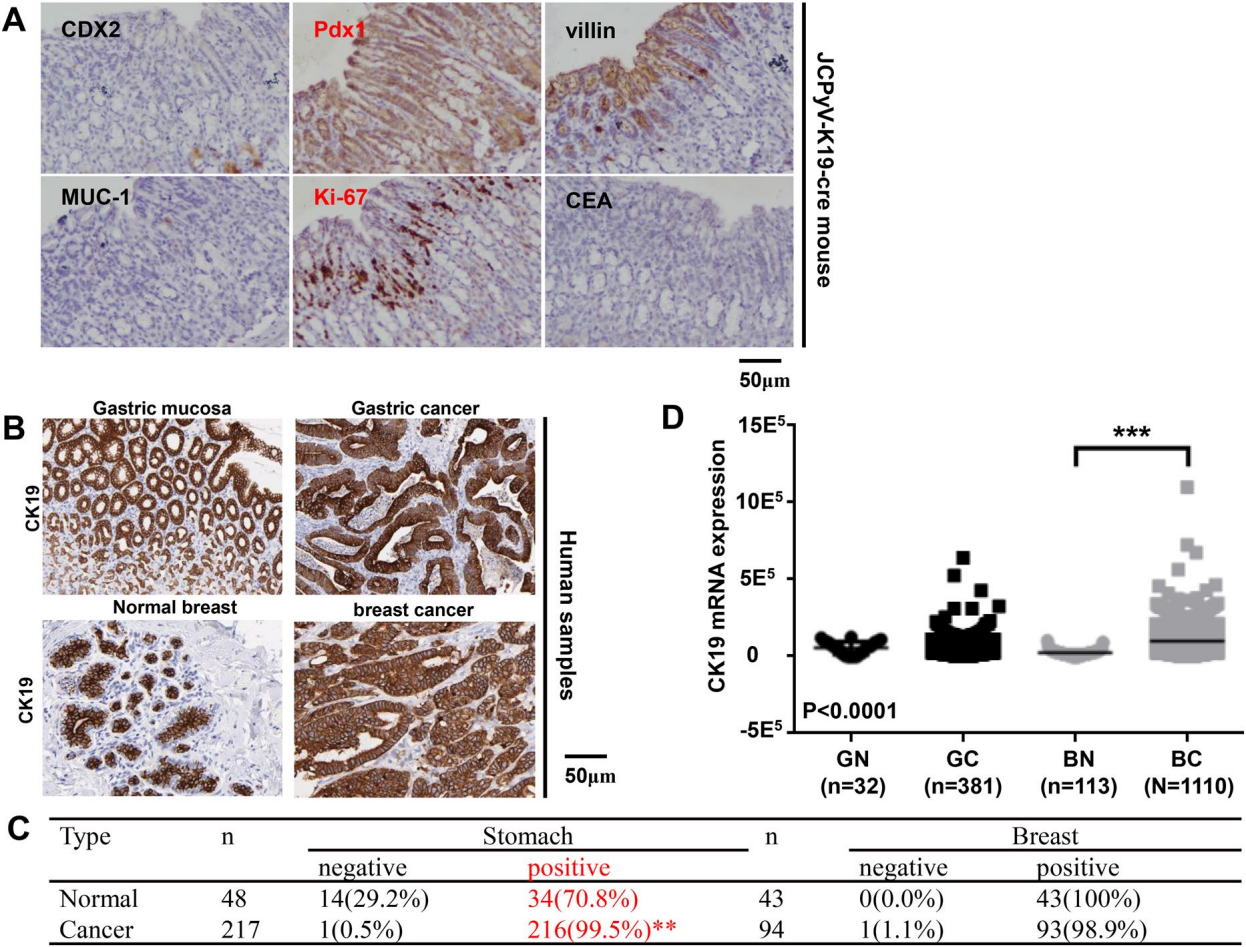
JCPyV T antigen was involved in gastric carcinogenesis. Tissue sections from a gastric poorly-differentiated carcinoma of a K19-cre/JCPyV T antigen transgenic mouse (♂, 18 months) were immunostained for CDX2, Pdx1, villin, MUC-1, ki-67, and CEA (**A**). CK19 expression was examined in gastric and breast cancers, and matched normal glands (**B**) as summarized in the table (**C**). The TCGA database was employed to analyze CK19 mRNA expression in gastric and breast cancers, and their corresponding normal tissues (**D**). BC, breast cancer; BN, breast normal tissue; CDX2, caudal type homeobox 2; CEA, carcinoembryonic antigen; CK19, cytokeratin 19; GC, gastric cancer; GN, gastric normal tissue; MUC1, mucin 1; Pdx1, pancreas/duodenum homeobox protein 1; TCGA, The Cancer Genome Atlas

**Fig. 3:**
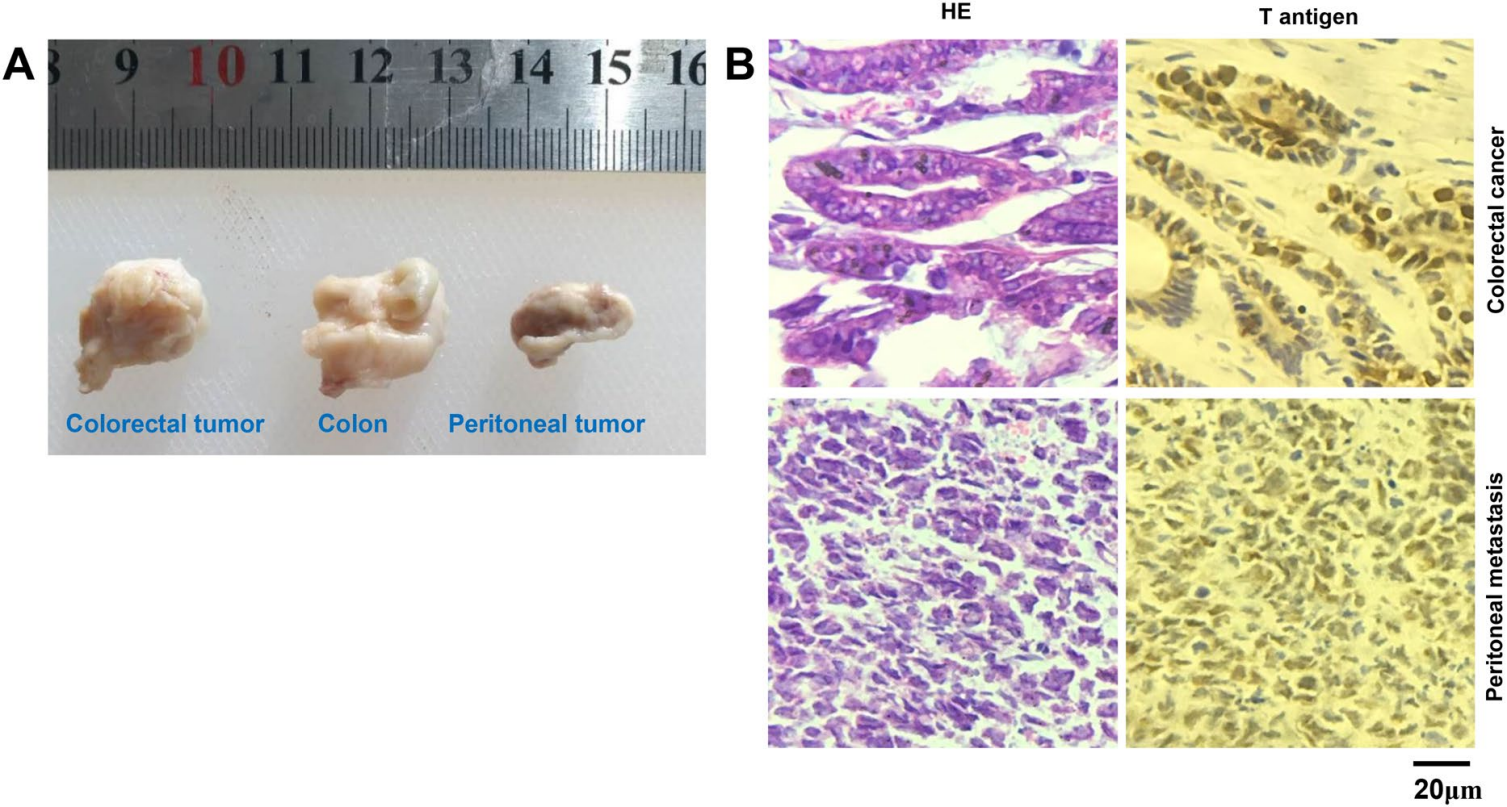
JCPyV T antigen was involved in colorectal carcinogenesis and progression. Colorectal cancer and peritoneal metastasis were observed in the transgenic mouse with villin-cre/JCPyV t antigen (♂, 10 months) both grossly (**A**) and using HE staining and immunostaining of T antigen (**B**). HE, hematoxylin and eosin

Grossly and histologically, we did not find gastric lesions in Atp4b-cre/T antigen and Capn8-cre/T antigen mice (data not shown), but we found a poorly differentiated carcinoma in K19-cre/T antigen (37.5%, 3/8) mice at 16–19 months of age according to HE staining (Table 2). Immunohistochemically, the positive expression of Pdx-1 (responsible for the early development of the pancreas and plays a major role in glucose-dependent regulation of insulin gene expression), ki-67 (cellular proliferation marker), and villin (the major microfilament-associated protein of the intestinal microvillus) were noted in spontaneous tumors of the JCPyV T antigen/K19-cre mouse. However, CDX2 (caudal type homeobox 2 responsible for intestinal inflammation and tumorigenesis) and MUC1 (a membrane-bound mucin involved in cell adhesion and invasion) were not expressed in the gastric lesions (Fig. 2A). To check for CK19 (an intermediate filament protein responsible for the structural integrity of epithelial cells) promoter activity, we examined CK19 protein and mRNA expression in human cancerous and matched normal tissues. Higher CK19 immunoreactivity was noted in human gastric cancer compared to matched mucosal tissues. However, a difference was not observed in CK19 expression between human breast cancer and matched normal tissues (Fig. 2B, C). The converse was true for CK19 mRNA expression according to human samples of the TCGA database (Fig. 2D). In villin-cre/T antigen mice, colorectal tumors and peritoneal metastatic foci were grossly observed (Fig. 3A). Primary and metastatic cancer cells showed T-antigen overexpression (Fig. 3B) with wild-type mice used as a negative control and lens tumors from alpha-crystallin-JCPyV T antigen as a positive control (data not shown). Tumor incidence was 44.4% (8/18) in villin-cre/T antigen mice at 9–10 months of age (Table 3).

**Table 2:** The age and sex distribution of spontaneous gastric cancer in K19-cre^+/−^; JCPyV T antigen^+/−^ mice

Number	Sex	Gastric cancer (months)
1	♂	–
2	♂	18
3	♂	–
4	♂	18
5	♂	16
6	♀	–
7	♀	–
8	♀	–

**Table 3:** The age and sex distribution of spontaneous colorectal cancer in villin-cre^+/−^; JCPyV T antigen^+/−^ mice

Number	Sex	Colorectal cancer (months)
1	♂	–
2	♂	–
3	♂	–
4	♂	–
5	♂	–
6	♂	–
7	♂	–
8	♂	10
9	♂	9
10	♂	9
11	♂	9
12	♂	9
13	♂	9
14	♀	–
15	♀	–
16	♀	–
17	♀	10
18	♀	9

### Hepatocellular carcinoma was observed in T-antigen transgenic mice

**Fig. 4:**
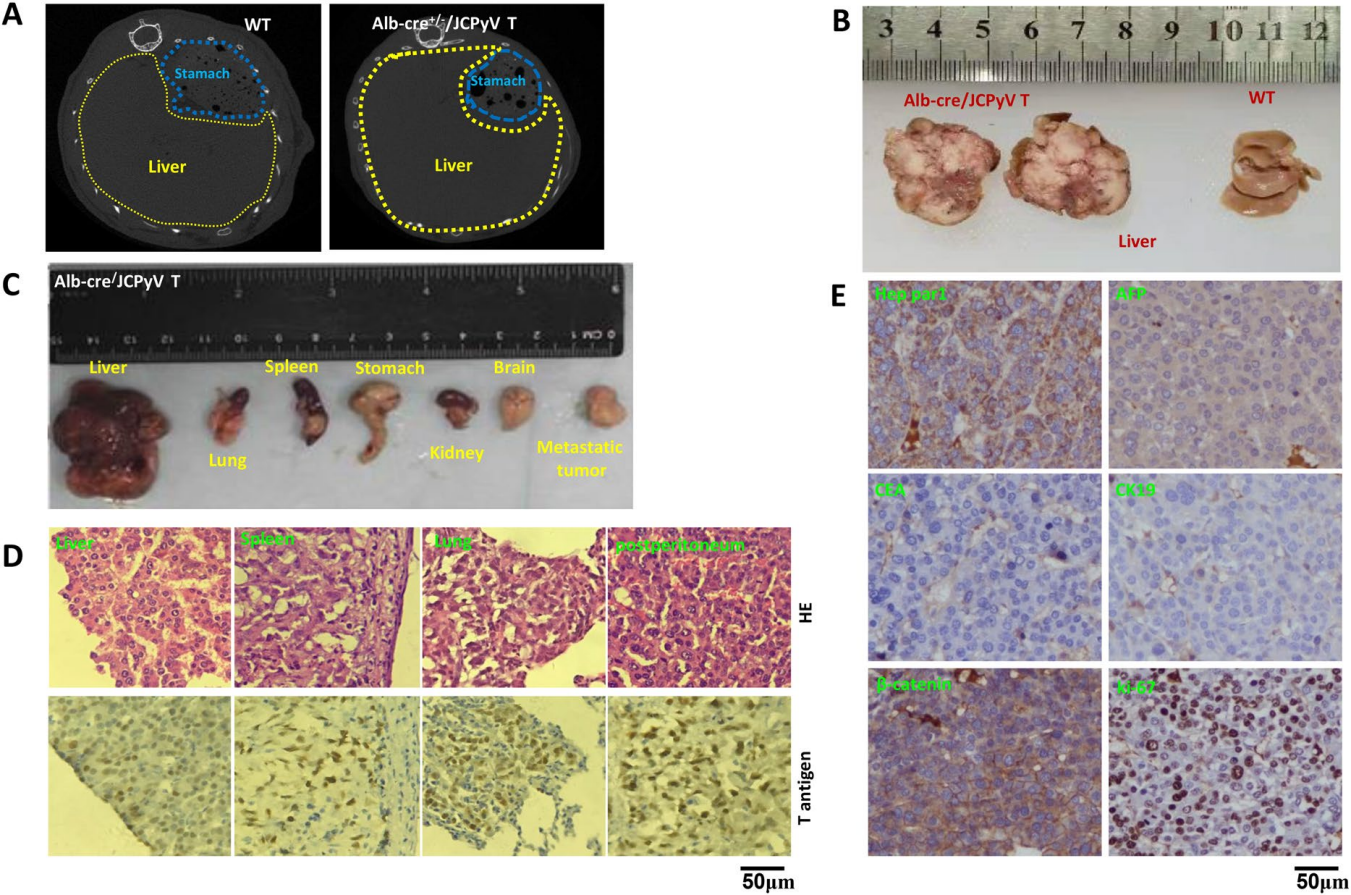
JCPyV T antigen–mediated hepatocellular carcinogenesis and progression. The livers from albumin-cre/JCPyV T antigen (♀, 7 months) and wild-type mice were examined by CT scanning (**A**) and grossly (**B**). The livers, lungs, spleens, stomachs, kidneys, brains, and a post peritoneal metastatic tumor were dissected from Alb-cre/T antigen mice (**C**). We found primary hepatocellular carcinoma and metastatic carcinoma in the spleen, lung and post- peritoneum according to HE staining and the immunostaining of T antigen (**D**). Hepatocellular carcinoma expressed Hep Par1, AFP, β-catenin and ki-67, but did not express CEA and CK19 (**E**). AFP, α-fetoprotein; CEA, carcinoembryonic antigen; CK19, cytokeratin 19; CT, computed tomography; HE, hematoxylin and eosin; T, T antigen; WT, wild-type

After CT scanning (Fig. 4A), we found diffuse swelling in the liver and ascites in Alb-cre/T antigen mice but not in wild-type mice. Grossly, necrosis was found in large liver tumors (Fig. 4B). We grossly (Fig. 4C) and histologically (Fig. 4D) found primary liver cancers, and metastatic cancers in the spleen, lung, and post-peritoneum, which showed strong T-antigen expression, while the stomach, kidney, and brain were normal. In liver tumors, α-fetoprotein (AFP; a marker for hepatocellular carcinoma), Hep Par1 (Hepatocyte paraffin 1, carbamoyl phosphate synthetase I, a marker protein in mitochondria of hepatocytes), β-catenin, and ki-67 proteins were found to be expressed. However, the expression of CEA (a cell adhesion protein anchored to the cell membrane by either a glycosylphosphatidylinositol moiety or a proteinaceous transmembrane and cytoplasmic domain, and highly expressed on many different cancers) and CK19 was not noted (Fig. 4E) suggesting that the primary liver tumors were hepatocellular carcinoma. Tumor incidence was 100.0% (13/13) in Alb-cre/T antigen mice at 3–10 months of age (Table 4).

**Table 4:** The age and sex distribution of spontaneous hepatocellular carcinoma in Alb-Cre^+/−^; JCPyV T antigen^+/−^ mice

Number	Sex	Hepatocellular carcinoma (months)
1	♂	3
2	♂	5
3	♂	5
4	♂	3
5	♂	8
6	♀	9
7	♀	7
8	♀	7
9	♀	7
10	♀	7
11	♀	10
12	♀	8
13	♀	8

### Pancreatic and gastrointestinal tumors were detectable in T-antigen transgenic mice

**Fig. 5:**
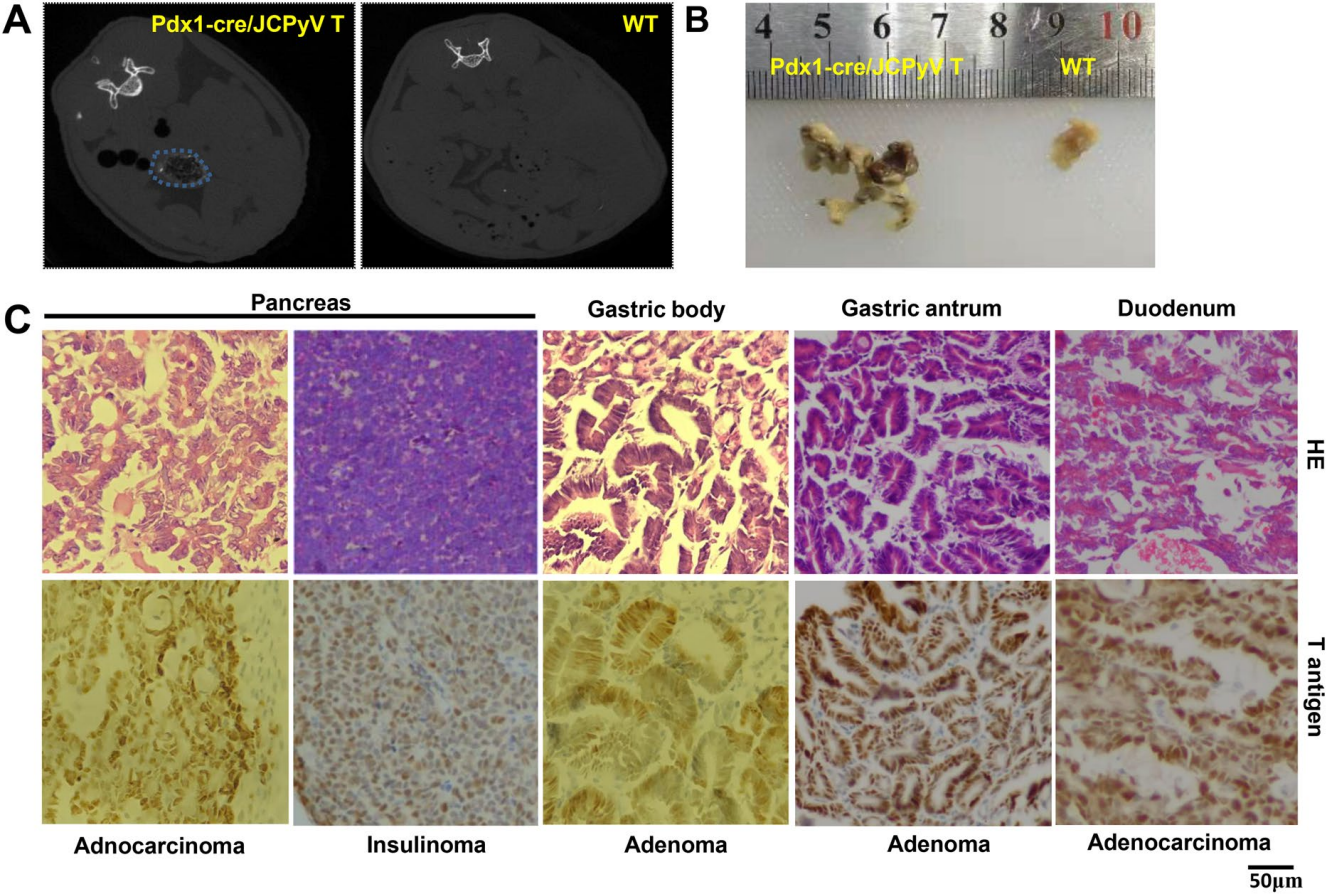
Multiple tumorigeneses were detectable in Pdx1-cre/JCPyV T antigen mice. According to CT scanning (**A**) and gross appearance (**B**), an irregular pancreatic tumor was found in Pdx1-cre/T antigen transgenic mouse (♂, 3 months) compared to wild-type mice. We found pancreatic ductal carcinoma and insulinoma, adenoma in the gastric body and antrum, and duodenal adenocarcinoma according to HE staining and immunostaining of T antigen (**C**). CT, computed tomography; HE, hematoxylin and eosin; WT, wild-type

According to CT images (Fig. 5A), pancreatic tumors with necrosis and grossly irregular tumors were observed in Pdx1-cre/T antigen mice (Fig. 5B). In each pancreatic tumor, we observed a pancreatic ductal cancer that was CEA positive (data not shown), and an insulinoma that was insulin positive and CEA negative (data not shown). Additionally, we found adenoma in the gastric body or antrum and adenocarcinoma in the duodenum (Fig. 5C). The incidences of gastric adenoma, and pancreatic and duodenal adenocarcinoma were 100% (15/15) in Pdx1-cre/T antigen mice, and the incidence of insulinoma was 16.7% (3/18) at the age of 2–5 months (Table 5).

**Table 5:** The age and sex distribution of spontaneous tumors in Pdx1-cre^+/−^; JCPyV T antigen^+/−^ mice

Number	Sex	Pancreatic cancer (months)	Insulinoma	Gastric adenoma	Duodenal adenocarcinoma
1	♂	2	−	+	+
2	♂	3	−	+	+
3	♂	3	−	+	+
4	♂	3	−	+	+
5	♂	3	−	+	+
6	♂	3.5	−	+	+
7	♂	3.5	+	+	+
8	♂	3	−	+	+
9	♀	2.5	−	+	+
10	♀	2	+	+	+
11	♀	2.5	−	+	+
12	♀	3	+	+	+
13	♀	3	−	+	+
46	♀	3	−	+	+
14	♀	3	−	+	+
15	♀	5	−	+	+

### Gastric, colorectal, and breast cancers were found in PGC-positive cells of transgenic mice expressing T antigen

**Fig. 6:**
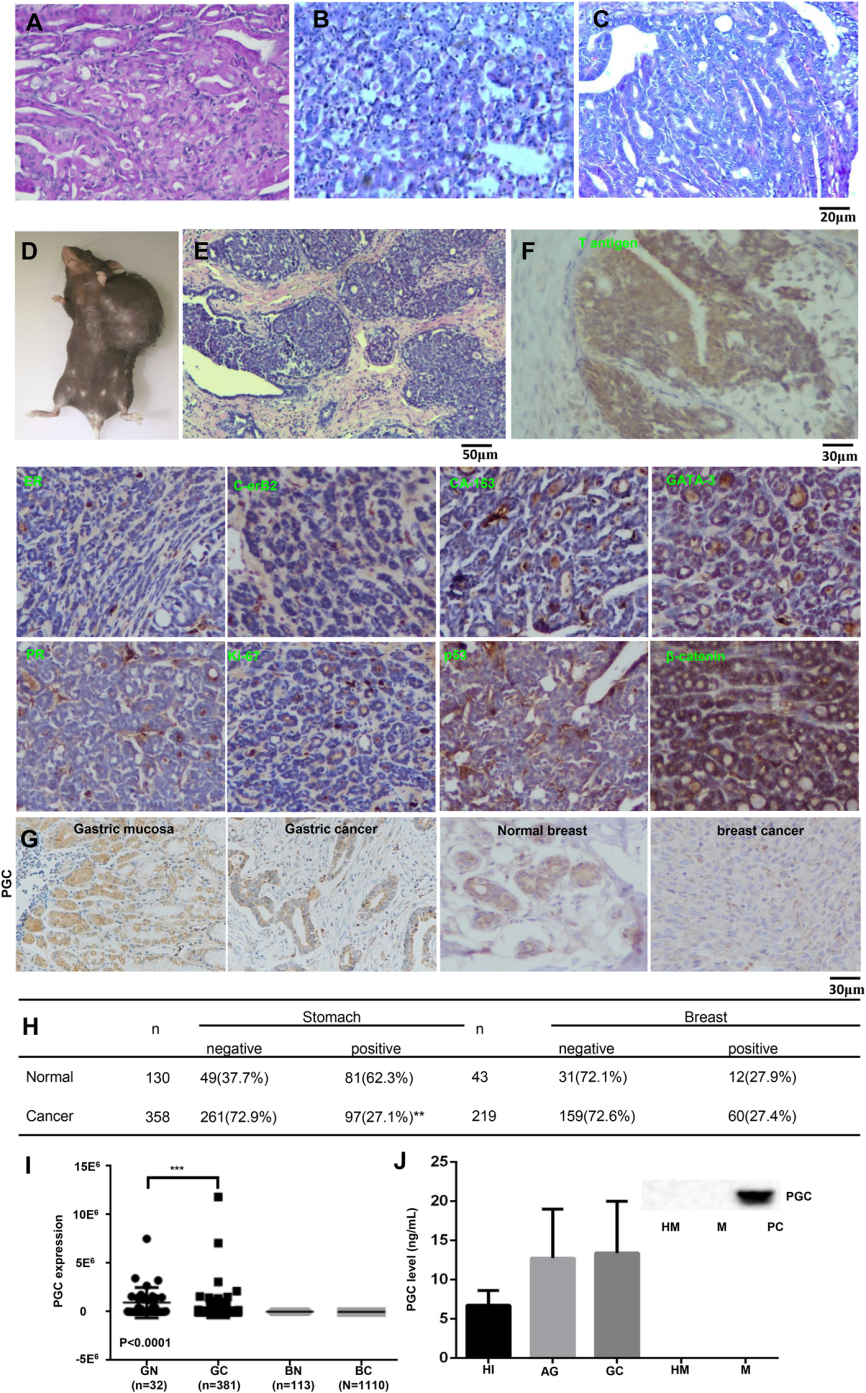
Multiple tumorigeneses were found in PGC-cre/JCPyV T antigen mice. Gastric cancer (**A**, ♀, 12 months; **B**, ♂,17 months) was histologically found in the PGC- cre/JCPyV T antigen transgenic mouse. Colorectal well-differentiated adenocarcinoma (**C**, ♂, 17 months) was also seen in transgenic mouse of PGC-cre/JCPyV T antigen. Breast tumor was grossly observed in the belly of the PGC-cre/JCPyV T antigen transgenic mouse (♀ 12 months, **D**). After HE staining (**E**) and immunostaining (**F**), lobular carcinoma of the breast was found to express T antigen, GATA-3, CA153, and β-catenin. The expression of PGC was immunohistochemically examined in gastric and breast cancers, and matched normal glands (**G**) as outlined in the table (**H**). The TCGA database was employed to analyze PGC mRNA expression in gastric and breast cancers, and their corresponding normal tissues (**I**). Finally, PGC content was determined by ELISA or western blot in the serum of a healthy individual, atrophic gastritis, gastric cancer, and human and bovine milks (**J**). AG, atrophic gastritis; BC, breast cancer; BN, breast normal tissue; CA153, carbohydrate antigen 153; ELISA, enzyme-linked immunosorbent assay; ER, estrogen receptor; GC, gastric cancer; GN, gastric normal tissue; HE, hematoxylin and eosin; HI, healthy individual; HM, human milk; M, milk powder; PC, positive control; PGC, pepsinogen C; PR, progesterone receptor; TCGA, The Cancer Genome Atlas

Histologically, we found gastric (Fig. 6A, B, 12.5%, 2/16) and colorectal (Fig. 6C, 6.25%, 1//16) adenocarcinomas in 17-month-old PGC-cre/T antigen mice (Table 6). Grossly, breast cancer was found in the abdominal area (Fig. 6D). Histologically, lobular carcinoma of the breast was observed in 37.5% (6/16) of PGC-cre/T antigen mice at 12–15 months of age (Fig. 6E). Immunohistochemically, the hyperexpression of T antigen, GATA-3 (a zinc-finger transcription factor specific for breast cancer), carbohydrate antigen 153 (specific biomarker for breast cancer), and β-catenin was noted. However, we found no expression of estrogen receptor, progesterone receptor, c-erB2, ki-67, and p53 in breast cancer (Fig. 6F) indicating that the breast tumors are triple-negative breast cancer. The expression of PGC was weaker in gastric cancer than in normal mucosa at both mRNA and protein levels according to bioinformatics analysis and immunostaining, respectively. However, a difference in PGC expression between breast cancer and normal tissue at both mRNA and protein levels was not found (Fig. 6G–I). Additionally, no PGC I was detectable in human and bovine milk, but a comparatively high level was found in healthy individuals, atrophic gastritis, and gastric cancer (Fig. 6J).

**Table 6:** The age and sex distribution of spontaneous tumors in PGC-Cre^+/−^; JCPyV T antigen^+/−^ mice

Number	Sex	Breast cancer (months)	Gastric cancer (months)	Colorectal cancer (months)
1	♀	–	–	–
2	♀	–	–	–
3	♀	–	–	–
4	♀	–	–	–
5	♀	–	–	–
6	♀	–	–	–
7	♀	–	–	–
8	♀	12	–	–
9	♀	11.5	–	–
10	♀	12.5	–	–
11	♀	12	–	–
12	♀	15	–	–
13	♀	15	–	–
14	♂	–	17	–
15	♂	–	17	–
16	♂	–	–	17

## Discussion

Cellular malignant transformation requires a series of genetic or epigenetic accumulations, including oncogene activation or overexpression. Although JCPyV is a highly neurotropic virus that induces brain tumors (Lee and Langhoff 2006), JCPyV DNA was found in respiratory and gastrointestinal tracts, and even in tonsil B lymphocytes and renal tubules due to JCPyV persistence (White and Khalili 2004; Zheng et al. 2007a, b). Therefore, establishing spontaneous tumors in transgenic mice expressing JCPyV T antigen in a tissue-specific manner is essential and helpful for understanding the potential oncogenic roles of JCPyV in epithelial carcinogenesis. In the present study, we generated CAG-LacZ-T antigen loxp/loxp mice to cross with various cre tool mice, in which cre is expressed in gastric pit, chief, parietal, intestinal, and pancreatic cells, as well as islet β cells and hepatocytes, respectively.

The K19 promoter was used to overexpress cyclooxygenase-2 and membrane binding prostaglandin e synthetase 1 in transgenic mice with gastric dysplasia (Itadani et al. 2009). Here, we found no gastric carcinogenesis in transgenic mice expressing T antigen in pit and parietal cells, while we observed gastric adenocarcinomas in K19- or PGC-cre/T antigen mice, and gastric adenoma in Pdx1-cre/T antigen mice. However, we could not elucidate the cell population responsible for gastric carcinogenesis among Pdx1-positive cells. Strangely, lung adenoma and adenocarcinoma, but not gastric neoplasms, were detected in K19-T antigen mice as described in our previous report (Noguchi et al. 2013). Additionally, K19-cre/PTEN ^f/f^ mice developed breast cancer, but not gastric cancer (Zhao et al. 2017). We also found no difference in K19 mRNA expression between gastric mucosa and cancer tissues, but higher expression in breast cancer than in normal tissue. As a result, a change in K19 promoter activity during carcinogenesis might possibly account for its tissue specificity and for its genetically spontaneous carcinogenesis.

Kane et al. (2020) observed the transition of colorectal sessile serrated lesions to cancer in BRAFV600E/villin-cre mice. Czéh et al. (2010) found that villin-cre × LoxP-SV40 T antigen mice developed intestinal and colorectal adenocarcinomas at 6 months of age. Intestinal adenomas or adenocarcinomas were generated in villin-cre + /JNK1^f/f^, Prss8^f/f^, PTEN^f/f^, and Msh2^f/f^ mice, but spontaneous serrated lesions, goblet cell hyperplasia, low-grade and high-grade dysplasias, and colorectal mucinous adenocarcinomas were seen in villin-cre/Notch-1^f/f^ mice (Tong et al. 2007; Kucherlapati et al. 2010; Byun et al. 2011; Dunkin et al. 2018; Bao et al. 2019). A meta-analysis indicated that the presence of JCPyV in colorectal tissues increased the odds ratio of colorectal cancer 4.70 times as much compared to normal mucosa (Shavaleh et al. 2020). Here, we first observed spontaneous colorectal adenocarcinomas in villin-cre/T antigen and PGC-cre/T antigen mice, indicating the potential oncogenic role of JCPyV T antigen in colorectal carcinogenesis.

Ochiai et al. (2020) found spontaneous hepatocellular carcinogenesis in Alb-cre/LSL- KrasG12D mice. Sekine et al. (2011) demonstrated that Alb-cre; Ctnnb1^f/f^ mice gained efficient deletions of β-catenin in hepatocytes at 2 months, but the reappearance and expansion of β-catenin–positive hepatocytes were seen with aging. In 12-month-old mice, pericentral hepatocytes were proportionally replaced with β-catenin–expressing hepatocytes, whereas most periportal hepatocytes appeared negative for β-catenin expression. Most 1-year-old mice spontaneously developed β-catenin–positive hepatocellular adenomas and carcinomas. In Alb-cre/T antigen mice, we found hepatocellular carcinoma (HCC) and peritoneal spreading as evidenced by ascites, CT scanning, and HE staining. Strong T-antigen expression was found in HCC, supporting the hypothesis that T-antigen protein had a potential oncogenic effect on hepatocellular carcinogenesis. Although T-antigen expression might be gradually decreased, the speed of HCC onset was faster than the weakening activity of the Alb promoter during hepatocellular carcinogenesis.

Feldmann et al. (2011) generated Pdx1-cre; Brca2^f/f^ and Pdx1-cre; Brca2^f/f^; LSL-Trp53 mice, and found pancreatic intraepithelial neoplasia. Pancreas-specific KrasG12D mice sufficiently developed pancreatic intraepithelial neoplasia using a Pdx1 promoter, and active Akt1 cooperated with *KrasG12D* to accelerate pancreatic carcinoma onset and progression (Albury et al. 2015; Ueda et al. 2019). Here, we also employed Pdx1-cre to activate T antigen and observed pancreatic ductal adenocarcinoma and insulinoma. The Pdx1 protein was widely expressed in gastric epithelial and intestinal mucosae such that Pdx1-cre/T antigen mice also suffered from gastric adenoma and duodenal adenocarcinoma. However, it is very difficult to speculate which cells are responsible for the histogenesis of these tumors due to a lack of cellular specificity of the Pdx1 promoter.

Mammary tumors arose in 30% of mouse mammary tumor virus (MMTV)-PIK3CA-H1047R mice and 13% of MMTV-PIK3CA-E545K mice (Merino et al. 2017). Guillory et al. (2010) developed a breast cancer model from a polyomavirus middle T antigen transgenic mouse model. Tzeng et al. (1993) established whey acidic protein-SV-T antigen transgenic mice, which developed mammary carcinomas with high frequency. It was of interest to note that the activation of T antigen in PGC-positive cells caused breast triple-negative lobular carcinoma in female transgenic mice, in line with PGC-cre/PTEN^f/f^ (E et al. 2023) and K19-cre/PTEN^f/f^ mice (Czéh et al. 2010), but gastric and colorectal cancer in male mice. Therefore, we speculated that the serum estrogen level, or the proliferation of lobular glands during breeding, might contribute to breast carcinogenesis in PGC-cre/T antigen transgenic mice.

Hachana et al. (2012) detected JCPyV T antigen DNA in invasive ductal carcinomas (28/112) but not in five invasive lobular carcinomas or six medullary carcinomas. However, we found breast triple-negative lobular adenocarcinoma in PGC-cre/T antigen mice although PGC was mainly expressed in gastric chief cells. Therefore, we believe that the PGC promoter might possibly be specific for the breast lobule, and any genetic alteration in the breast lobule might result in breast cancer. Based on our existing knowledge, it is reasonable to speculate that a mother’s breast might functionally substitute for an infant’s stomach due to the following causes: (1) breast milk and gastric digestion supply nutrients for the human body; (2) no PGC production and similar pH value (6.84) of breast milk and infant serum; (3) similar anatomic structure between breast and stomach: the presence of myocytes around glands in spite of different germ layer origins (ectoderm vs. endoderm); (4) both sucking and digestive secreting reflexes include neuroendocrine regulation; and (5) hereditary diffuse gastric cancer carries a *CDH1* mutation, and displays gastric and breast cancer (Shenoy 2019).

If a virus has an oncogenic role, it must infect cells and subsequently encode oncogenic proteins to replicate DNA, disrupt cell function, and finally to induce carcinogenesis. According to our prior findings (Zheng et al. 2022, 2021), we found JCPyV copies that differed according to tissue types (stomach < lung < breast < liver < pancreas) since the distinct distribution of its receptors (α2, 6-linked sialic acid, non-sialylated glycosaminoglycans and serotonin) determined its different infectivities (Elphick et al. 2004; Gee et al. 2006; Geoghegan et al. 2017). Nukuzuma et al. (2016) found a suppressive effect of the topoisomerase I inhibitors, topotecan and β-lapachone, on JCPyV propagation in human neuroblastoma cells. The adipocyte plasma membrane protein, phosphoinositide 4 kinase γ, and its regulatory subunit, PIK3R5, promoted JCPyV infection in human glial cells (Clark et al. 2020; Haley et al. 2020). Uleri et al. (2011) have shown that splicing factor 2/alternative splicing factor (SF2/ASF) negatively regulated transcription and splicing of JCPyV genes via direct interaction with the viral promoter in glial cells. The hyperexpression of SF2/ASF induced growth and proliferation of JCPyV-transformed tumor cells, and either endogenous or ectopic liver-inhibitory isoform expression mediated the degradation of T antigen in a JCPyV-transgenic mouse tumor cell line (Bellizzi et al. 2015). Transcriptionally, we found that alternative splicing of the T-antigen intron did not show tissue specificity in the stomach, pancreas, liver, and intestine, in line with our previous study (Gou et al. 2015). Transmembrane receptors, cytoplasmic signal proteins, and T-antigen protein status might result in the tissue specificity of its oncogenesis in a JCPyV-related cancer.

In conclusion, JCPyV T antigen might induce gastrointestinal tumorigenesis with cell specificity that is not linked to alternative splicing of the T-antigen intron. The combination of T antigen, exposure to cyclic estrogen or the proliferation of lobular glands during breeding might initiate breast lobular adenocarcinogenesis in PGC-positive cells. These spontaneous tumor models are good cancer models that will allow us to investigate the potential oncogenic role of JCPyV T antigen and possible novel therapeutic approaches to cancer.

## Data Availability

No data are required to be deposited. The datasets generated and/or analyzed during the current study are available from the corresponding author on reasonable request.
